# A New Sperm Concentration Threshold for Y Chromosome Microdeletion Analysis in Infertile Men: Could It Be Azoopermia?

**DOI:** 10.5152/tud.2024.24061

**Published:** 2024-05-01

**Authors:** Ali Çift, Can Benlioğlu, Mehmet Özgür Yücel, Muhammer Özgür Çevik, Bedreddin Kalyenci, Alper Gök, Sait Sever, Hasan Sulhan, Haydar Bağış, Ali Ayyıldız

**Affiliations:** 1 Department of Urology, Adıyaman University Faculty of Medicine, Adıyaman, Türkiye; 2 Department of Medical Genetics, Adıyaman University Faculty of Medicine, Adıyaman, Türkiye; 3 Department of Urology, University of Health Sciences, Etlik City Hospital, Ankara, Türkiye; 4 Department of Urology, Adıyaman Education and Research Hospital, Adıyaman, Türkiye

**Keywords:** Male infertility,, Y chromosome microdeletion,, azoospermia factor,, cost

## Abstract

**Objective:**

We aimed to assess the frequency of Y-chromosome microdeletions (YCMs) in a non-multiethnic urban population in our region, define predictive factors, and determine a new clinical threshold for YCMs in infertile men.

**Materials and Methods:**

A total of 281 patients with a sperm concentration ≤5 million/mL were retrospectively evaluated. Oligozoospermic and/or azoospermic patients with a sperm concentration of ≤5 million/mL were screened for the YCM analysis.

**Results:**

Y-chromosome microdeletion was detected in 9 (3.2%) of the 281 patients. All patients with YCM were azoospermic. The presence of azoospermia, a high follicle-stimulating hormone level, and a high luteinizing hormone level were found to be important determinants for the identification of a microdeletion (*P* = .002, *P* = .002, and *P* = .021, respectively). If the presence of azoospermia and a sperm concentration threshold of <1 million/mL had been applied for the YCM test, the number of tests performed would have been reduced by 54.4% (153 tests) and 42.7% (120 tests), respectively, resulting in cost saving of approximately $11 474 and $9000, respectively.

**Conclusion:**

We recommend that the threshold for sperm concentration for YCM analysis be set at <1 million in individuals in developed countries and only in patients with azoospermia in developing countries, in order to reduce costs and save labor by excluding unnecessary tests. These proposed thresholds (azoospermia and sperm counts less than <1 million/mL) provide cost-effectiveness by significantly reducing the number of genetic tests ordered without affecting the diagnosis rate.

**Main Points**

- Y-chromosome microdeletion was detected in 9 (3.2%) of the 281 patients. All patients with YCM were azoospermic.
- If the presence of azoospermia and a sperm concentration threshold of <1 million/mL had been applied for the YCM test, the number of tests performed would have been reduced by 54.4% (153 tests) and 42.7% (120 tests), respectively, resulting in a cost saving of approximately $11 474 and $9000, respectively.
- We recommend that the threshold for sperm concentration for YCM analysis be set at less than 1 million in individuals in developed countries and only in patients with azoospermia in developing countries, in order to reduce costs and save labor by excluding unnecessary tests.

## Introduction

Among genetic abnormalities in the etiology of male infertility, Klinefelter syndrome and Y-chromosome microdeletion (YCM) are the leading causes.^1^ The relationship between YCM and spermatogenesis was first identified in 1976 by Tiepolo and Zuffardi. As a result of their study, the authors named the fertility gene or gene regions as the azoospermia factor (AZF) located on the Y chromosome.^2^ Azoospermia factor genes are located in the Y chromosome’s long arm in the AZFa, AZFb, and AZFc regions. These deletions can be complete, partial, and occur in more than one region. The AZFa microdeletion is the least common (5%) while the AZFc microdeletion is the most common (65-70%). Sperm cannot be found in patients with AZFa, AZFb, AZFb+c, and AZFa+b+c microdeletions. Therefore, there is no need for micro testicular sperm extraction (mTESE). When mTESE is performed in azoospermic patients with AZFc microdeletion, 50-75% of sperm can be obtained.^3^

Identifying men with YCM in advance can assist in clinical decision-making before starting invasive procedures. Since this genetic defect can be passed on to boys, couples are recommended to seek genetic counseling.

The threshold to be used as an indication for screening YCM in infertile men has not yet been precisely determined. As stated in many guidelines, YCM may be found in all infertile men with sperm concentrations less than 5 million/mL.^4,5^ A study reported that all patients with YCM consisted of oligozoospermia patients with a sperm concentration of <2 million/mL.^6^ In a meta-analysis performed by Kohn et al,^7^ in oligozoospermic patients with a sperm concentration of >1-5 million/mL, the prevalence of YCM was reported as 0.8%.^7^ However, the current European Association of Urology (EAU) guideline notes that while an absolute threshold for clinical testing cannot be universally given, patients may be offered testing if sperm counts are <5 million/mL, but must be tested if <1 million/mL.^1^ The indications for YCM testing are still being debated in the published literature.

In this study, we aimed to formulate a more cost-effective clinical threshold for ordering YCM genetic testing by considering the prevalence of YCM and other predictive factors in a non-multiethnic urban population of infertile men.

## Material and Methods

Local Ethics Committee approval (2020/11-6) was received. Informed consent was waived as the study was retrospective. A total of 1150 patients who were followed up with a diagnosis of male infertility in our clinic between January 2015 and January 2020 were retrospectively evaluated. The study included 281 male infertile oligozoospermic and/or azoospermic patients with a sperm concentration of less than 5 million/mL, who had complete records, were sexually active, and were not able to achieve pregnancy spontaneously within 1 year despite not using contraception. Medical history was obtained for all patients, and a physical examination and 2 semen analyses were performed. Follicle-stimulating hormone (FSH), luteinizing hormone (LH), and total testosterone (TT) levels were evaluated. All semen samples were analyzed according to the criteria specified in the 2010 guidelines of the World Health Organization.^8^ Patients with obstructive azoospermia were excluded. Oligozoospermic patients with a sperm concentration of ≤5 million/mL were screened for the YCM analysis.

**Figure 1.**
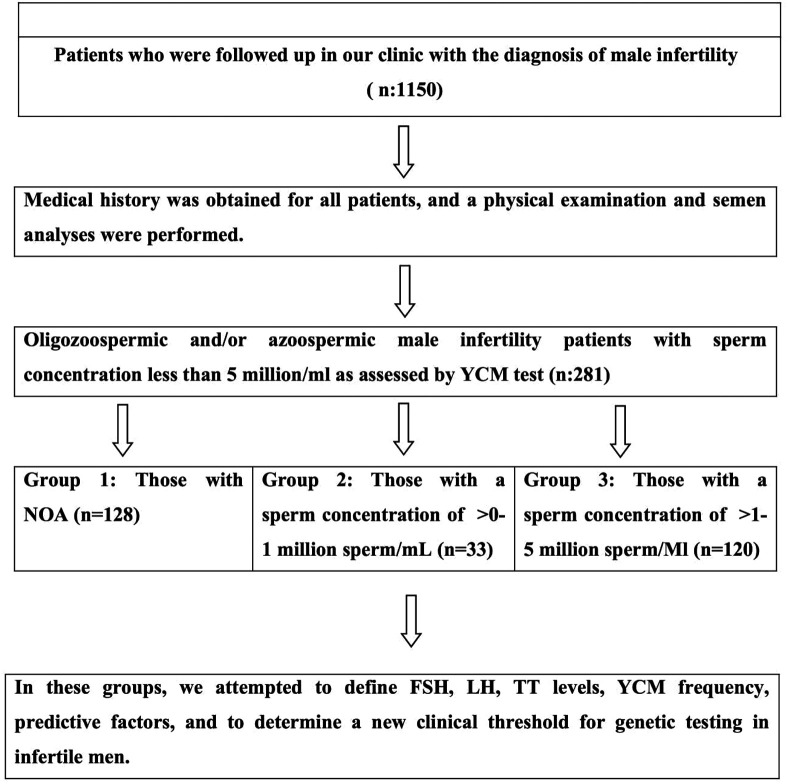
Flow chart to represent the data collection process visually.

The patients were evaluated in 3 groups: (1) those with NOA; (2) those with a sperm concentration of >0-1 million sperm/mL; and (3) those with a sperm concentration of >1-5 million sperm/mL. In these groups, FSH, LH, TT levels, and YCM frequency were evaluated, and an attempt was made to define predictive factors in these parameters and to determine a new clinical threshold for genetic testing in infertile men. A flow chart visually depicting the data collection process is shown in Figure 1.

### Genetic Testing

Genetic testing for YCMs was performed according to the EAA/European Molecular Genetics Quality Network (EMGQ) guidelines.^4^

### Statistical Analysis

Statistical Package for Social Sciences v. 20 (SPSS/IBM®, Chicago, Ill, USA) software package was used for statistical analyses. Levene’s test for equality of variances and Q-Q plot tests were used to determine the data’s conformity to normal distribution. Box plots were used to present the values and relationships between AZF deletions and age, FSH, LH, TT, and semen analysis and between semen analysis and age, FSH, LH, TT, and AZF deletions. The Student’s *t*-test was used for the comparative analysis of parametric data between the groups, and the Kruskal–Wallis and Mann–Whitney *U*-tests were employed for non-parametric data. The Spearman and Pearson tests were conducted for the correlation analysis. *P* < .05 was considered statistically significant.

## Results

**Figure 2.**
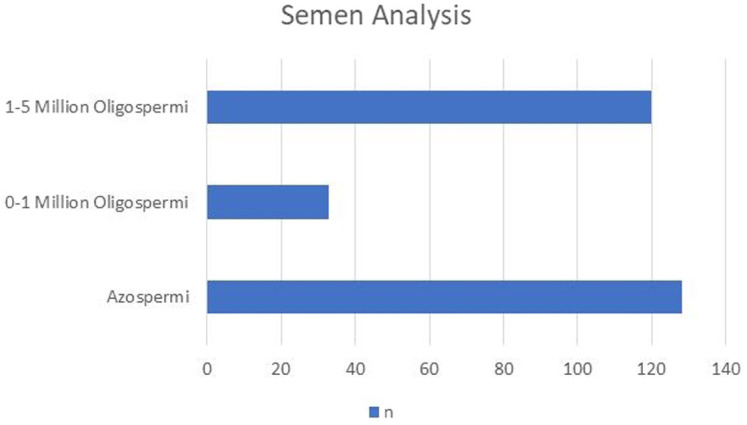
Distribution of semen analysis in the study population.

**Figure 3.**
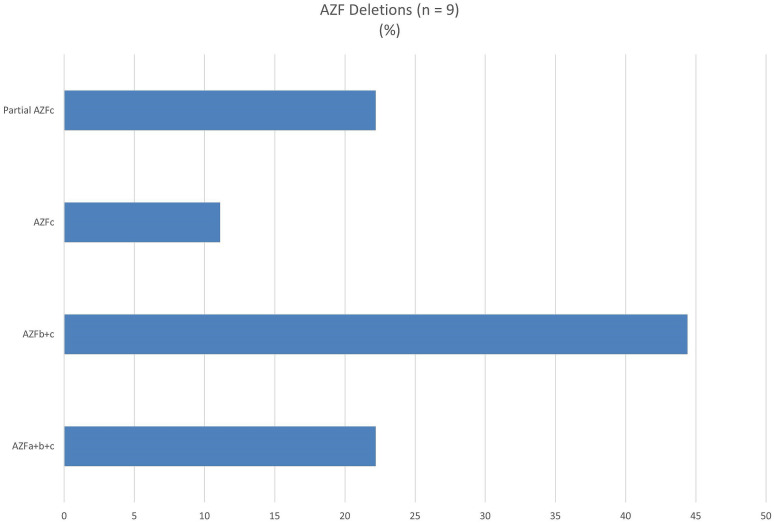
Distribution of AZF microdeletions in the study population.

**Figure 4.**
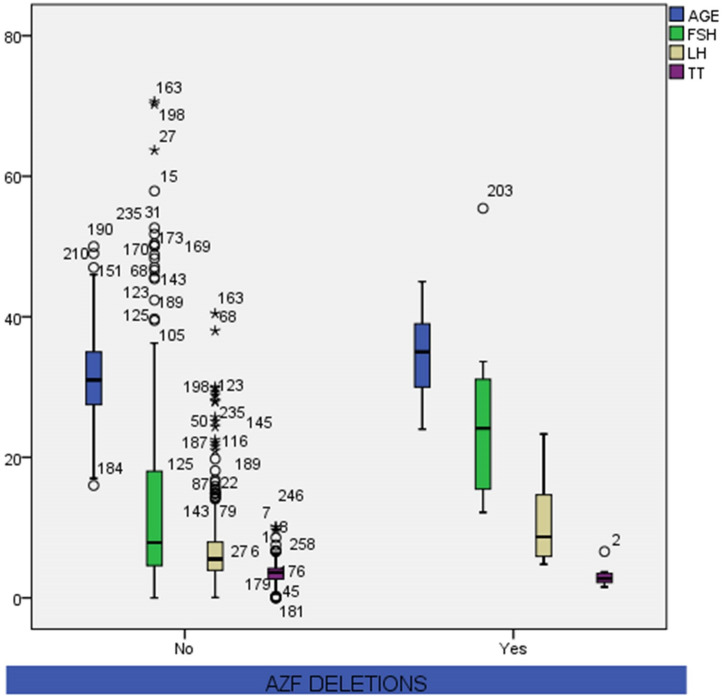
Y-chromosome microdeletion predictors.

One thousand one hundred fifty patients were evaluated for male infertility. The sperm concentration of 281 (24.4%) of these patients was ≤5 million/mL. The mean age of these patients was 31.7 ± 6.11 (16-50) years. The rates of patients in group 1, 2, and 3 were 45.55% (n = 128), 11.75% (n = 33), and 42.70% (n = 120), respectively (Figure 2). Y-chromosome microdeletion was detected in 9 (3.2%) of the 281 patients. The characteristics of these patients are presented in Table 1. When the distribution of YCMs was evaluated in the study population, AZFb+c was the most common microdeletion (44.44%), followed by microdeletions of the AZFc partial (22.22%), AZFa+b+c (22.22%), and AZFc complete (11.11%) regions. The microdeletion of the AZFa and AZFb region alone was not observed (Figure 3). All the patients with YCM were azoospermic, and YCM was not detected in any of the oligozoospermic patients. The presence of azoospermia, a higher FSH level, and a higher LH level were found to be important predictors of microdeletion (*P* = .002, *P* = .002, and *P* = .021, respectively). The TT level did not predict the presence of a microdeletion (Tables 2 and 3) (Figure 4). If the presence of azoospermia and a sperm concentration threshold >0-1 million/mL had been applied for the YCM test, the number of tests performed would have been reduced by 54.4% (153 tests) and 42.7% (120 tests), respectively, resulting in a cost saving of approximately $11 474 and $9000, respectively (Table 4). Table 5 summarizes the characteristics of previous studies in which oligozoospermic patients were not detected to have YCM.

## Discussion

It has been reported that the global prevalence of YCM in infertile men varies between 1% and 55.5%^9^ and its prevalence in Europe is 1.8-13%.^10-14^ A similar prevalence range has been indicated in Turkish populations.^15-19^ It is considered that the reason for the varying prevalence of YCMs may be related to the differences in geographical regions where the studies were conducted, the ethnic origins of the participants, and methodologies used. Our results are similar to the studies in the literature.

When the studies in the literature were evaluated, no predictive value was found between YCM and commonly used parameters such as hormone levels, testicular size, varicocele, and infection.^20, 21^

Johnson et al^11^ reported that a higher FSH level and a lower sperm concentration were important determinants for defining a microdeletion, while the TT and LH levels and testicular volume were not effective in predicting the presence of a microdeletion. The authors also noted that the FSH level was highly variable and not clustered together, making it difficult and unreliable to determine a threshold based on this parameter. Therefore, in light of their study data, the authors did not recommend using the FSH level as a threshold to decide whether microdeletions should be tested.

Contrary to the studies in the literature, we found a negative correlation between the FSH and LH levels and sperm concentrations in our correlation analysis, while the relationship between these levels and YCM was minimal. However, the FSH and LH levels were very variable in the group with YCM; therefore, we do not recommend using either as a threshold for testing microdeletions.

Many guidelines state that a sperm concentration of less than 5 million/mL is required for YCM analysis.^1,22^ Giacco et al (2014) reported that all patients with YCM had a sperm concentration of less than 2 million/mL.^6^ In a meta-analysis, the frequency of YCM in patients with oligozoospermia >1-5 million/mL was reported as 0.8%. This rate is similar to the 0.7% frequency of YCM reported among oligozoospermic patients with more than 5 million/mL in the EAU guideline. Therefore, in the later evaluations of male infertility, guidelines are recommended using a screening threshold with a sperm concentration of less than 1 million/mL as an indication for YCM screening.^7^ In light of these studies, the recent EAU guideline published in 2020 states that YCM screening must be undertaken in patients with a sperm concentration of ≤1 million/mL, while it can be recommended for those with a sperm concentration of ≤5 million/mL.^1^

Ortac et al^23^ reported that the frequency of YCM was 3.8% in 3023 oligozoospermic patients with less than 5 million/mL. In addition, the frequency of YCM was reported to be 6.8% for patients with NOA (n = 1581), 1.0% for oligozoospermic patients with a sperm concentration of >0-1 million/mL (n = 799), and 0.15% for oligozoospermic patients with a sperm concentration of >1-5 million/mL (n = 643).

In our study, we found the prevalence of YCM to be 3.2% (9/281) in those with NOA. Our results are lower than those reported in most studies in the available literature. In our study, we did not find YCM in any of the oligozoospermic patients. Similar to our study, YCM was not found in any of the oligozoospermic patients in many studies.^24-30^ In 7 of the previous studies as well as our study, a total of 1233 patients with a sperm concentration of less than 5 million/mL were evaluated, and none were found to have YCM.

Ortac et al^23^ evaluated the sensitivity and specificity of the YCM test in azoospermic patients and those with a sperm concentration threshold of ≤1 million/mL and determined these values as 92.2% and 49.3%, respectively, in the former and 99.1% and 22.1%, respectively, in the latter. The authors also noted that if the accepted indications for the YCM test in hospitals had been the presence of azoospermia and a sperm concentration threshold of ≤1 million/mL, the number of tests would have been reduced by 1442 and 643, respectively, resulting in a saving of $108 150 and $48 223, respectively.

In a study by Johnson et al,^11^ the frequency of YCM was found to be 5.5%. All of the patients with YCM in this study had a sperm concentration of less than 0.5 million. The authors determined that at a sperm concentration threshold of 0.1 million/mL, the number of tests in their hospital would decrease by 24%, leading to a cost saving of £55 000.

In our study, the YCM test was only positive in azoospermic patients. When the literature was evaluated, similarly, YCM was not detected in any of the oligozoospermic patients in 7 studies (24-30). If we had used the presence of azoospermia and a sperm concentration threshold of ≤1 million/mL as an indication for the YCM test, the number of tests performed would have decreased by 54.4% (153 tests) and 42.7% (120 tests), respectively, and we would have saved approximately $11 474 and $9000, respectively. This result may have significant economic implications for countries like Türkiye. In Türkiye, the cost of the YCM test is about $75. YCM screening is an economically expensive test for developing countries. For this reason, cost-effectiveness should be taken into account in performing these tests. We recommend lowering the sperm concentration threshold for YCM analysis and performing it only in patients with azoospermia. This result constitutes a strength of our study. In line with our results, the presence of azoospermia can be considered a new indication for the YCM analysis since it would provide cost-effectiveness by significantly reducing the number of tests performed (153/281, 54.4%).

This study’s potential limitations include its retrospective design, moderate sample size, and a relatively localized population from a single center of an isolated geographical region.

## Conclusion

The polymerase chain reaction-based YCM analysis is a very suitable approach for the diagnosis, management, and counseling of infertile men. However, it is costly. We recommend that the threshold for sperm concentration for YCM analysis be set at less than 1 million in individuals in developed countries and only in patients with azoospermia in developing countries, in order to reduce costs and save labor and increase the chance of diagnosis by excluding unnecessary tests. These proposed thresholds (azoospermia and sperm counts less than <1 million/mL) provide cost-effectiveness by significantly reducing the number of genetic tests ordered without affecting the diagnosis rate. In addition, as the hormone levels were found to be very variable in the group with YCM, we do not recommend using their thresholds for the decision to test for microdeletions. Our data will be useful in adopting appropriate genetic counseling methods and assisted reproduction techniques in infertility clinics. Our results should be supported by larger-scale and multi-center studies.

## Figures and Tables

**Table 1.** Demographic and Clinical Characteristics of the Patients with Y-Chromosome Microdeletion

	Age (Years)	FSH (mIU/mL)	LH (mIU/mL)	TT (ng/mL)	Semen Analysis (n = 9)	AZF Deletion
	36	15.48	8.67	3.45	Azoospermia	AZFc (Komplet)
	41	14.9	5.5	6.59	Azoospermia	AZFc (partial)
	39	33.64	14.67	3.72	Azoospermia	AZFc (partial)
	24	12.18	5.9	3.16	Azoospermia	AZFb+c
	45	31.11	17.96	2.75	Azoospermia	AZFb+c
	30	55.43	23.33	2.2	Azoospermia	AZFb+c
	35	24.13	10.84	2.26	Azoospermia	AZFb+c
	29	18.5	6.7	1.55	Azoospermia	AZFa+b+c
	31	25.4	4.79	2.18	Azoospermia	AZFa+b+c
Mean	34.4 ± 6.69	25.6 ± 13.40	10.09 ± 6.44	3.09 ± 1.48		

AZF, azoospermia factor; FSH, follicle-stimulating hormone; LH, luteinizing hormone; TT, total testosterone; YCM, Y-chromosome microdeletion.

**Table 2. Y-chromosome Microdeletion:** Predictors

YCM^*^	Microdeletions, mean ± SD; n	No Microdeletions, mean ± SD; n	*P*
Sperm concentration (million/mL)	0 ± 0.00; 9	1.037 ± 0.05; 272	.002
FSH^*^ level (mIU/mL)	25.64 ± 4.46; 9	13.38 ± 0.80; 272	.002
LH^*^ level (mIU/mL)	10.92 ± 2.14; 9	7.29 ± 0.36; 272	.021
TT^*^ level (ng/mL)	3.09 ± 0.49; 9	3.48 ± 0.09; 272	.179

FSH, follicle-stimulating hormone; LH, luteinizing hormone; TT, total testosterone; YCM, Y-chromosome microdeletion.

**Table 3.** Spearman’s Correlation Analysis

Correlation Analysis (AZF Deletion-Sperm Concentration/FSH/LH/TT)^*^		
	Correlation Coefficient	*P*
Sperm concentration	−0.189	.001
FSH level	0.186	.002
LH level	0.138	.020
TT level	−0.080	.180

FSH, follicle-stimulating hormone; LH, luteinizing hormone; TT, total testosterone.

**Table 4.** Cost Saving with the Use of Different Sperm Concentrations as an Indication for Y-Chromosome Microdeletion Screening

Sperm Concentration Threshold, million/mL	n	Total Cost ($)^*^	Reduction in Tests (%) (n)	Total Cost Saving ($)^*^
Azoospermia	128	9600	54.4 (153)	11 474
>0-1 million sperm/mL	33	2475	42.7 (120)	9000
>1-5 million sperm/mL	120	9000	-	-

^*^$, American dollars. Estimated cost of the YCM test: $75/patient.

**Table 5.** Characteristics of Literature Studies in Which Y-Chromosome Microdeletion Was Not Detected in Oligozoospermic Patients

			>0-1 million sperm/mL		>1-5 million sperm/mL	
Authors (Year)	Country	Criteria for Identifying Complete YCM	Complete YCM	Men Screened (n)	Complete YCM	Men Screened (n)
Kovacheva et al (2018)	Bulgaria	EAA/EMQN criteria	0	46	0	46
Khurana et al (2014)	USA (Ohio)	Deletion of all regional STSs	0	205	0	205
Tzschach et al (2001)	Germany	Deletion of all regional STSs	0	64	0	64
Calleja Macías et al (2003)	Mexico	Undefined by authors deletion of sY254, sY255 applied	0	16	0	16
Gruber et al (2003)	Austria	Deletion of all regional STSs	0	154	0	154
Ioulianos et al (2002)	Cyprus	Undefined by authors deletion of sY254, sY255 applied	0	48	0	48
Friel et al (2001)	Ireland	Deletion of all regional STSs	0	7	0	7
Current study	Türkiye	EAA/EMQN criteria	0	33	0	120
**Total**			0	573	0	660

EAA, European Academy of Andrology; EMQN, European Molecular Genetics Quality Network; YCM, Y-chromosome microdeletion.
